# Psychological mechanisms linking AI hallucinations to user trust and behavioral intentions toward AI-generated videos: an S–O–R perspective

**DOI:** 10.3389/fpsyg.2026.1781974

**Published:** 2026-03-27

**Authors:** Jun Liu, Yue Sun, Kaiqi Xiao

**Affiliations:** 1 College of Art, Cheongju University, Cheongju, Republic of Korea; 2 School of Design, Fujian University of Technology, Fuzhou, China; 3 College of Media, Tonghua Normal University, Tonghua, China

**Keywords:** AI hallucination, AI-generated video, behavioral intention, perceived trust, stimulus–organism–response model, uncanny valley eeriness

## Abstract

With the widespread adoption of AI-generated videos in media content production, their visual credibility and the associated issues of user trust have attracted increasing attention. Although AI-generated videos have become progressively more realistic, user-perceived visual anomalies (AI hallucinations) may elicit negative psychological responses, undermine trust, and ultimately shape behavioral intentions. Grounded in the Stimulus–Organism–Response (S–O–R) framework, this study systematically examines how AI hallucinations influence perceived trust through uncanny valley eeriness and perceived realism, and how these effects further translate into behavioral intention. We manipulated three levels of AI hallucination (low, medium, and high) in AI-generated video stimuli and recruited 408 participants to view and evaluate the videos. Hypotheses were tested using partial least squares structural equation modeling (PLS-SEM) and analysis of variance (ANOVA). The results indicate that AI hallucinations significantly increase uncanny valley eeriness and reduce perceived realism; both factors further affect behavioral intention via perceived trust, with perceived realism showing the strongest predictive effect on trust. Moreover, uncanny valley eeriness, perceived realism, perceived trust, and behavioral intention differed significantly across hallucination levels. These findings support the applicability of the S–O–R model in the context of viewing AI-generated videos and delineate a psychological transmission mechanism through which AI hallucinations shape trust judgments and behavioral responses via affective and cognitive processing, offering a new theoretical perspective on credibility construction in generative visual content.

## 1 Introduction

As artificial intelligence (AI) enters a new stage characterized by generative AI content (AIGC) (Dogan et al., 2023; Oke, 2008), it has profoundly reshaped the ecosystems of media production and digital creativity (Kietzmann et al., 2018; Liu and Sun, 2025; Whittaker et al., 2020; Yu and Zheng, 2023) and has become a central driving force behind content transformation (Pitonáková et al., 2025). Among these developments, video production stands at the forefront of this wave of technological innovation (Fan et al., 2023). A wide range of generative tools has not only substantially improved production efficiency (Khachatryan et al., 2023; Zhu et al., 2023), but also significantly lowered the barriers to creative entry, thereby accelerating the widespread adoption of AI-generated video (Yu et al., 2024).

However, gains in production efficiency have not automatically translated into broad user acceptance. The industry is currently confronting a pronounced “trust dilemma.” Although AI's technical capabilities in film and video production have advanced rapidly (Bougueffa Eutamene et al., 2024; Gioia et al., 2013; Ragot et al., 2020; Wu et al., 2020) and are widely regarded as powerful tools for expanding creative possibilities (Çalli and Alma Çalli, 2025; Tsiavos and Kitsios, 2025), users' psychological responses have not consistently reflected this technological progress. A growing body of research indicates that viewers often experience unease and skepticism when watching AI-generated videos (Ajder and Glick, 2021; Xu Z. et al., 2025). This highlights a critical tension: although AI-generated videos appear increasingly realistic, user trust may decline rather than increase in certain contexts (Song and Shin, 2024).

From a psychological perspective, addressing this trust crisis requires a close examination of how users perceive “visual anomalies” (Çalli and Alma Çalli, 2025). Given that generative AI systems still fall short of human performance in terms of fine-grained naturalness and semantic coherence (Pellas, 2023; Zhang et al., 2025a), users are often able to detect subtle visual deviations, such as facial distortions or discontinuities in motion (Aziz et al., 2025). In this study, these subjectively perceivable visual irregularities are collectively conceptualized as “AI hallucinations.” Prior research suggests that such anomalies can undermine users' evaluations of content credibility and subsequently influence their intention to use the technology (Aziz et al., 2025; Çalli and Alma Çalli, 2025; Zhou and Lu, 2025). When highly realistic videos reveal unnatural local details, they are likely to trigger the “uncanny valley” effect—characterized by feelings of eeriness and cognitive discomfort (Çalli and Alma Çalli, 2025). Although information distortion in AIGC has attracted scholarly attention, there remains a lack of systematic empirical investigation into how these distortions shape individuals' emotional responses, cognitive evaluations, and trust judgments (Sun et al., 2024).

To address this gap and to fundamentally explain the origins of the trust crisis surrounding AI-generated video, this study develops a theoretical framework grounded in the Stimulus–Organism–Response (S–O–R) model. Specifically, the intensity of AI hallucinations is conceptualized as the external stimulus (S); uncanny valley responses, perceived realism, and perceived trust are treated as organismic states (O); and behavioral intention is defined as the ultimate response (R). More specifically, this study seeks to answer the following research questions:

(1) In the context of AI-generated videos, how do AI hallucinations influence perceived trust through uncanny valley eeriness and perceived realism, and how do these effects further translate into behavioral intention?

(2) During the organism-stage psychological processing, what roles do uncanny valley eeriness, perceived realism, and perceived trust play in predicting behavioral intention, and how do they differ in relative importance?

(3) Do different levels of AI hallucinations lead to significant differences in uncanny valley eeriness, perceived realism, and perceived trust, thereby altering users' patterns of psychological responses?

This study carries significant theoretical and practical implications. At the theoretical level, it extends the application boundaries of AI hallucination and uncanny valley theories from the perspective of psychological processing, providing a structured explanatory framework for understanding how trust mechanisms are formed in AI-generated content. At the practical level, this research quantifies the specific psychological impact of visual anomalies on audiences. By identifying key factors that contribute to trust erosion, it offers empirical evidence to support trust-oriented AI content design and system optimization. To achieve these objectives, the study integrates experimental design with questionnaire-based surveys and employs structural equation modeling and analysis of variance to systematically test the proposed hypotheses and underlying mechanisms.

## 2 Literature review and hypothesis development

### 2.1 Theoretical foundation

The Stimulus–Organism–Response (S–O–R) model was originally proposed by Mehrabian and Russell (Manthiou et al., 2017) and represents a classic theoretical framework in environmental psychology for explaining how external stimuli influence behavioral responses through individuals' internal psychological processing (Cho et al., 2019; Mishra et al., 2022; Yang et al., 2021). This model emphasizes that individuals do not respond passively or directly to environmental stimuli; rather, external stimuli are transformed into concrete behavioral outcomes through a series of internal processes, including emotional experiences, cognitive evaluations, and psychological judgments (Mehrabian and Russell, 1974; Mladenović et al., 2023). Accordingly, the S–O–R model provides a systematic representation of the transmission pathway from “external stimulus–internal psychological state–behavioral response” and has been widely applied in research on consumer psychology (Islam et al., 2021; Zhu et al., 2020), media psychology and human–computer interaction (Liu and Sun, 2025), as well as immersive digital experience studies (Daassi and Debbabi, 2021; Kim et al., 2020).

Existing research has demonstrated that the S–O–R model has strong explanatory power in digital media and technology use contexts. Its core assumption—that external stimuli influence individuals' psychological states and thereby elicit behavioral responses—has been validated across a variety of virtual and digital experience settings (Hess et al., 2014). In prior studies, external stimuli are typically operationalized as system functional features, interface design elements, or informational cues (e.g., online reviews) (Cho et al., 2019; Kim et al., 2020; Zhou et al., 2023; Zhu et al., 2020). Organism-level responses are mainly reflected in psychological variables such as emotional experiences, perceived realism, and perceived trust (Hess et al., 2014; Liu and Sun, 2025), while behavioral responses are commonly expressed as usage intention, continuance intention, or recommendation behavior (Pham et al., 2024).

Within the context of AIGC and digital content generation, both content quality and presentation cues have similarly been shown to exert significant effects on users' psychological responses (Tan et al., 2025; Tao et al., 2025). Some studies indicate that the quality of AIGC content influences users' sense of empowerment and satisfaction, and that these organism-level psychological responses further determine continuance intention (Zhou and Ma, 2025). In addition, in digital visual environments, cue incongruity or reduced realism often has a pronounced impact on users' emotional arousal, authenticity evaluations, and trust judgments, and indirectly affects behavioral intention through emotional and cognitive mechanisms (Zhou et al., 2022). Taken together, these findings suggest that the S–O–R model is not only suitable for explaining the effects of general digital stimuli on behavior, but also effective in capturing the emotion–cognition–trust processing mechanisms triggered by visual cue anomalies.

Based on this theoretical foundation, the S–O–R model is highly compatible with the AI-generated video viewing context examined in this study. Specifically, AI hallucinations, as a form of visual cue incongruity, can undermine the structural consistency and semantic stability of generated content. Their stimulative effects on users' perceptual systems align with the theoretical definition of “stimulus” (S). Such visual deviations may induce psychological incongruence related to naturalness, biological plausibility, and controllability, thereby activating uncanny valley experiences at the emotional level, reducing perceived realism at the cognitive level, and weakening users' subjective evaluations of content credibility at the judgment level. Together, these emotional, cognitive, and evaluative responses constitute the organism-stage (O) psychological processing.

**Figure 1:**
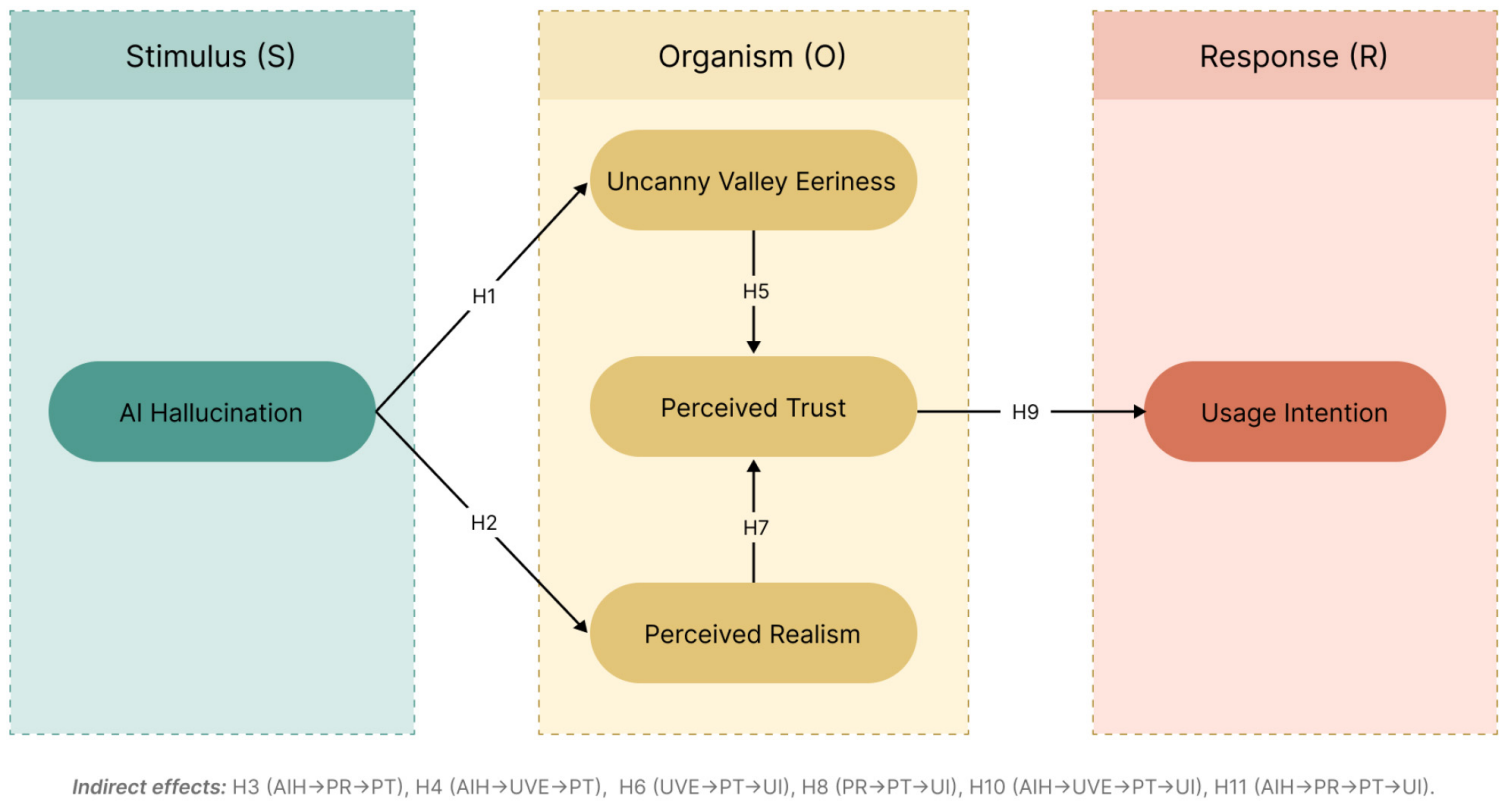
Structural model within the S–O–R framework.

Accordingly, this study adopts the S–O–R model as its overarching analytical framework (as shown in Figure 1). AI hallucination intensity is conceptualized as the stimulus (S), representing the degree of deviation in visual form, motion continuity, and semantic coherence in AI-generated videos. Uncanny valley eeriness, perceived realism, and perceived trust are incorporated as organism-level responses (O), capturing the emotion–cognition–trust processing chain experienced by users while viewing AI-generated videos. Behavioral intention is specified as the final response (R), reflecting users' tendencies to use, adopt, and disseminate AI-generated videos. Through this framework, the study aims to systematically reveal the mechanisms underlying users' psychological responses under different AI hallucination conditions and to further extend the application of the S–O–R theory in research on AIGC video credibility and human–computer interaction.

### 2.2 Hypothesis development

#### 2.2.1 AI hallucinations

AI hallucinations are commonly defined as outputs generated by artificial intelligence systems that deviate from reality or contain logical inconsistencies, and they are widely regarded as an important source of risk that undermines the credibility of generated content (Christensen et al., 2025; Kumar et al., 2023). From the user perception perspective, AI hallucinations essentially represent a form of “distorted generation,” manifested as logical errors, factual deviations, or fabricated content. Such distortions disrupt the logical consistency and semantic coherence between generated content and the real world, thereby significantly reducing users' perceived realism (Carrasco-Farré, 2022; Sun et al., 2024). Prior studies have shown that these “almost real but not truly real” outputs are likely to induce cognitive dissonance and psychological discomfort, which in turn activate negative emotional responses and weaken users' trust judgments (Sun et al., 2024; Zhou and Lu, 2025).

In the context of visual content generation, the psychological impact of AI hallucinations is particularly pronounced. When AI-generated videos exhibit distortions in morphological structure, motion continuity, or semantic alignment, such hallucinatory anomalies disrupt the correspondence between the content and the real world, thereby undermining users' sense of realism and credibility judgments (Aziz et al., 2025; Zhang et al., 2025b). Moreover, issues such as distorted facial structures, discontinuous movements, lip–speech asynchrony, erroneous body compositing, and illogical scene construction cause AI-generated videos to appear “highly realistic yet not fully real.” These characteristics readily evoke feelings of unease and emotional distancing, further triggering uncanny valley experiences and reducing trust levels (Duong and Vo, 2025; Weisman and Peña, 2021). Based on this reasoning, the present study argues that AI hallucinations, as perceptible visual anomaly cues, systematically influence users' psychological responses through affective and cognitive processing pathways. Accordingly, the following hypotheses are proposed:

**H1:** AI hallucinations have a positive effect on uncanny valley eeriness.

**H2:** AI hallucinations have a negative effect on perceived realism.

**H3:** AI hallucinations negatively affect perceived trust through perceived realism.

**H4:** AI hallucinations negatively affect perceived trust through uncanny valley eeriness.

#### 2.2.2 Uncanny valley eeriness

The concept of the “uncanny valley” was originally proposed to explain the discomfort and emotional distancing experienced by humans when encountering highly anthropomorphic artificial entities that contain subtle anomalies (Masahiro et al., 2012). Subsequent research has indicated that this effect is not limited to robots or virtual characters, but is broadly applicable to any visual stimulus that is “highly realistic yet not fully natural” (Tan et al., 2025). When AI-generated visual content closely resembles reality at an overall level but reveals unnatural or inconsistent details at a local level, users are likely to experience pronounced feelings of eeriness and psychological discomfort, thereby weakening their affective trust in the AI system or the content itself (Weisman and Peña, 2021).

In the context of AI-generated videos, such negative psychological reactions are equally salient. Research has shown that when AI videos display features that are “almost human but still unnatural” in visual or auditory aspects, users are more prone to feelings of unease, suspicion, and emotional distancing, which further reduces their trust in and acceptance of the content (Duong and Vo, 2025). In addition, when AI-generated videos achieve high visual realism but lack emotional naturalness and human-like qualities, viewers often experience typical uncanny valley responses, characterized by eeriness, emotional detachment, and cognitive unease. These reactions diminish both content acceptance and trust in the creator or system (Çalli and Alma Çalli, 2025). Accordingly, uncanny valley eeriness is considered a key emotional mechanism through which AI visual stimuli influence users' trust judgments and behavioral intentions. Based on the above analysis, the following hypotheses are proposed:

**H5:** Uncanny valley eeriness has a negative effect on perceived trust.

**H6:** Uncanny valley eeriness negatively affects behavioral intention through perceived trust.

#### 2.2.3 Perceived realism

Perceived realism refers to users' subjective judgments regarding the extent to which visual content aligns with the real world in terms of logical structure, visual presentation, contextual consistency, and physical principles (Chivileva et al., 2023). In research on generative content, realism is widely recognized as a key cognitive factor influencing users' evaluations of information quality and credibility (de Ridder, 1996). The degree of visual fidelity—particularly features such as detail representation, texture quality, and lighting naturalness—often directly shapes users' perceptions of authenticity and reliability (Luo et al., 2025).

Existing studies indicate that the closer AI-generated images appear to reality, the more likely users are to perceive the content as trustworthy (Aziz et al., 2025). This pattern also applies to AI-generated videos. Prior research has shown that AI-generated instructional videos often reduce audience trust due to insufficient realism in facial expressions, movement coordination, or speech naturalness, thereby confirming the critical role of visual realism in trust formation (Xu T. et al., 2025). Furthermore, when evaluating the credibility of AI-generated videos, users typically rely on realism cues such as visual naturalness, the coherence of movements and expressions, and narrative logic. When content is perceived as more realistic and more consistent with everyday experience, trust and acceptance tend to increase accordingly (Duong and Vo, 2025). Based on this reasoning, the following hypotheses are proposed:

**H7:** Perceived realism has a positive effect on perceived trust.

**H8:** Perceived realism positively affects behavioral intention through perceived trust.

#### 2.2.4 Perceived trust

Perceived trust refers to users' subjective judgments and positive expectations regarding a technological system's ability, reliability, integrity, and benevolence under conditions of uncertainty, and it is widely regarded as a critical psychological mechanism influencing technology adoption behavior (Zhou and Lu, 2025). In AI-driven human–computer interaction contexts, trust not only shapes users' evaluations of the accuracy and stability of system outputs, but also serves as an important psychological link between perceived quality and behavioral intention, playing a central role in technology acceptance processes (Afroogh et al., 2024; Choung et al., 2023; Hu et al., 2025). Prior research has shown that when users believe an AI system can consistently provide reliable, stable, and user-benefiting outputs, they are more likely to develop trust and demonstrate stronger intentions to use and adopt the technology (Yu et al., 2025).

In the domain of AI-generated content, trust is likewise a key psychological factor determining whether users accept, share, or use AI-generated videos (Gillath et al., 2021). Further empirical evidence suggests that when users perceive AI-generated videos as credible and capable of delivering stable and reliable informational experiences, they are more inclined to continue viewing or engaging with such content, resulting in higher acceptance and continuance intentions (Lao et al., 2025). Specifically, when AI-generated short videos are perceived as credible, reliable, and sufficiently authentic in terms of visual, narrative, or emotional cues, users' perceived trust increases significantly, thereby promoting subsequent behaviors such as continued use, attention, or participation (Duong and Vo, 2025). Accordingly, the following hypothesis is proposed:

**H9:** Perceived trust has a positive effect on behavioral intention.

#### 2.2.5 Multistage psychological processing hypotheses within the S–O–R framework

Based on the Stimulus–Organism–Response (S–O–R) model, this study integrates the above hypotheses to construct a multistage psychological processing mechanism through which AI hallucinations influence users' behavioral intentions. As AI hallucinations function as external visual stimuli that simultaneously elicit users' emotional responses (uncanny valley eeriness) and cognitive evaluations (perceived realism), and as these responses further shape behavioral intention through trust judgments (perceived trust), this study posits that AI hallucinations do not directly affect user behavior. Instead, their influence on behavioral responses is indirect, operating through multiple organism-level psychological processing stages. On this basis, the following integrated pathway hypotheses are proposed:

**H10:** AI hallucinations negatively affect behavioral intention by eliciting uncanny valley eeriness and reducing perceived trust.

**H11:** AI hallucinations negatively affect behavioral intention by reducing perceived realism and, in turn, perceived trust.

## 3 Methodology

### 3.1 Overview of the research design

This study aims to examine how different levels of AI hallucinations influence users' behavioral intentions toward AI-generated videos by shaping their emotional responses (uncanny valley eeriness), cognitive responses (perceived realism), and psychological judgments (perceived trust). Grounded in the Stimulus–Organism–Response (S–O–R) framework, the study systematically manipulates AI hallucination levels (low, medium, and high) using AI video generation technologies and produces corresponding experimental stimuli for viewing and measurement.

**Figure 2:**
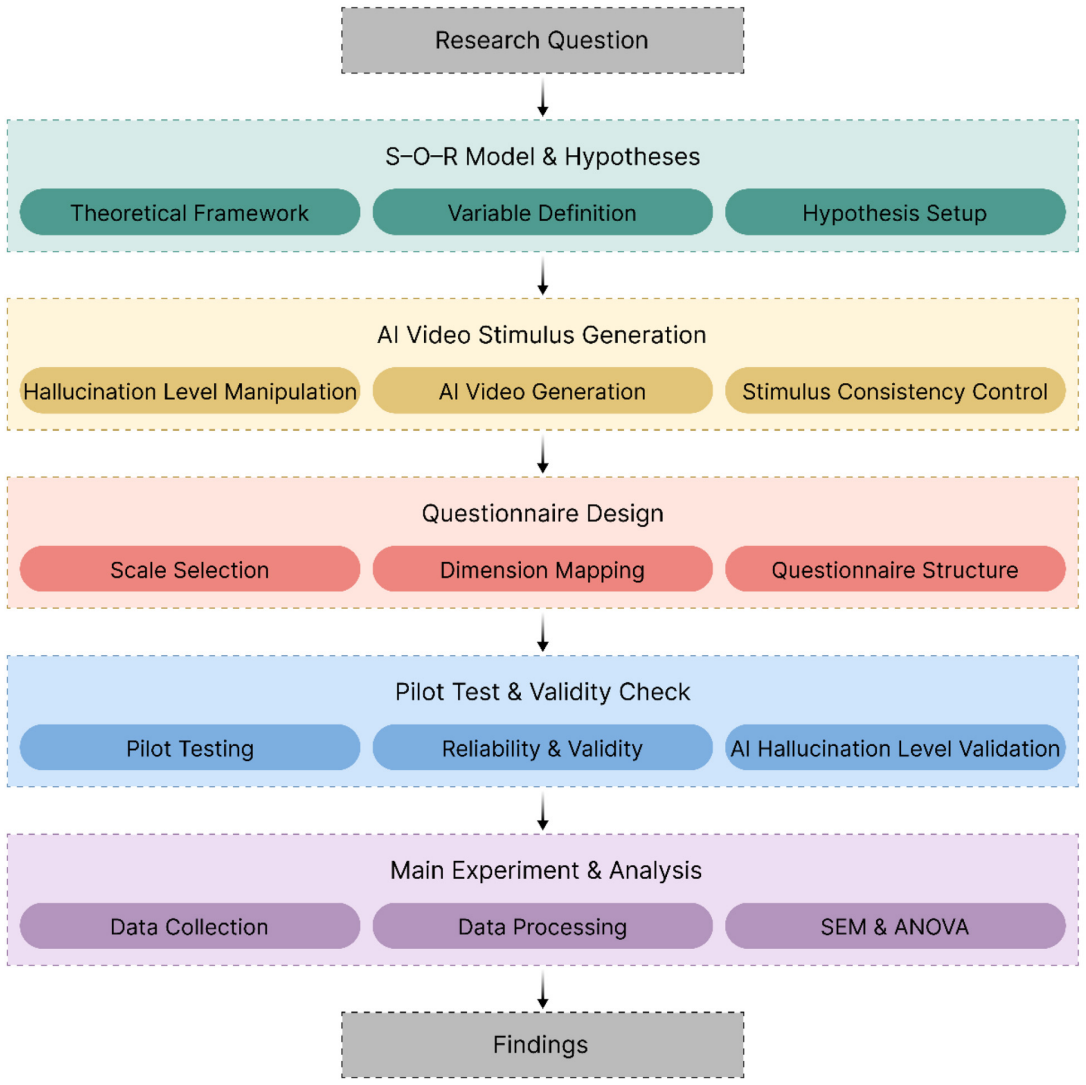
Flowchart of research methodology.

As illustrated in Figure 2, the overall research procedure consists of the following steps: (1) constructing a theoretical framework based on the S–O–R model and formulating research hypotheses; (2) generating video stimuli with varying levels of AI hallucinations using AI video generation systems; (3) designing a measurement questionnaire to quantitatively assess AI hallucinations, uncanny valley eeriness, perceived realism, perceived trust, and behavioral intention; (4) conducting a pilot study to evaluate the reliability and validity of the stimuli and measurement scales, as well as the feasibility of the experimental procedure; and (5) collecting formal samples and empirically testing the hypotheses using partial least squares structural equation modeling and analysis of variance.

Within this experimental framework, variations in AI hallucination intensity can be clearly mapped onto users' emotional, cognitive, and trust-related responses, enabling the quantitative identification of the pathways through which AI hallucinations influence behavioral intention. This approach provides systematic empirical evidence and model-based support for understanding trust formation mechanisms in AI-generated video contexts.

### 3.2 Experimental design

#### 3.2.1 Stimulus materials and video generation

In this study, AI hallucination intensity (low, medium, and high) was treated as the external stimulus variable (S). AI hallucinations mainly manifest as structural and semantic distortions in generated videos, including abnormal object forms, discontinuous movements, and incoherent narratives or logic. To ensure robust manipulation and result reproducibility, a set of video stimuli representing three levels of hallucination intensity (low, medium, and high) was created based on the same video theme and visual context for experimental presentation.

All video stimuli were produced using an image-to-video generation approach. During the generation process, a unified generation script, character configuration, and camera layout were applied across all videos. Model version, lighting style, composition ratio, and scene background were kept consistent to minimize the influence of extraneous variables. Manipulation of AI hallucination intensity was achieved solely through differential prompt settings related to semantic details and motion descriptions, while the narrative theme, character setup, and camera structure remained identical. This design ensured that hallucination intensity constituted the only systematic difference among the stimuli. All videos were generated with identical output parameters (24 fps, 1,080 p resolution, 5-s duration, and a 16:9 aspect ratio) to ensure comparability across stimulus conditions.

The three final video stimuli were evaluated by five experts from the fields of visual communication, digital media, and human–computer interaction. The experts conducted a consistency review to verify the perceptual discriminability of different hallucination levels and to confirm that low, medium, and high hallucination conditions were clearly distinguishable at the perceptual level while maintaining comparable overall visual quality. Following expert validation, the three videos were finalized as the experimental stimuli.

#### 3.2.2 Questionnaire and experimental procedure

In the formal experiment, all participants viewed three AI-generated videos corresponding to low, medium, and high AI hallucination conditions. After watching each video, participants completed the corresponding questionnaire items. By manipulating only AI hallucination intensity while keeping video content and visual style consistent, the study ensured that participants' judgments were primarily based on variations in visual anomaly severity, thereby minimizing potential confounding effects arising from content differences.

It is important to clarify that the questionnaires in this study were primarily distributed and administered through university campuses—particularly within design- and media-related departments—as well as through relevant online digital media communities. This sampling strategy inevitably resulted in a certain concentration in the demographic characteristics of the final sample, which consisted predominantly of individuals aged 18–24, women, and participants with academic backgrounds related to visual media.

However, this specific group represents a typical population of “digital natives” and serves as early adopters and prospective core professionals in the field of AIGC visual tools. They constitute one of the most central target audiences for current AI video generation technologies. Due to their frequent exposure to visual generative content in both academic training and professional practice, they demonstrate heightened sensitivity to subtle structural distortions and semantic discontinuities. Consequently, data collected from this group objectively reflect authentic user responses during real-world interactions with AIGC tools, thereby exhibiting a high level of ecological validity. This context provides a particularly rigorous and well-suited setting for examining the micro-level psychological transmission mechanisms proposed within the S–O–R framework.

More importantly, the within-subject design employed in this study ensured that each participant served as their own control. This methodological approach effectively minimized the influence of between-subject variance—such as differences in gender, age, and disciplinary background—on causal inferences regarding the core variables. As a result, the robustness of the mechanism testing was strictly maintained at the statistical level.

The questionnaire consisted of two sections: (1) demographic information and (2) 15 measurement items covering five latent variables—perceived AI hallucinations, uncanny valley eeriness, perceived realism, perceived trust, and behavioral intention (see Table 1). The measurement scales were developed based on prior research and tailored to the characteristics of AIGC visual content. They were further reviewed and refined by five experts in artificial intelligence, human–computer interaction, and design media to ensure conceptual clarity and structural validity. All items were measured using a 7-point Likert scale (1 = strongly disagree, 7 = strongly agree). Before completing the questionnaire, participants were explicitly instructed to base their ratings on whether the video appeared natural or incongruent, whether movements were smooth, and whether abnormal details were present, while minimizing the influence of personal aesthetic preferences or content interest.

**Table 1:** Questionnaire dimensions and specific content.

**Construct**	**Content**	**References**
AI hallucination	I noticed abnormalities in the facial structure shown in the video.	(Jin et al., 2025; Song et al., 2025), Expert interviews
	I noticed that the characters' facial features in the video did not conform to real-world physiological structures.	
	I noticed clear visual distortions or misalignments in this video.	
Uncanny valley eeriness	While watching, I felt that the characters' appearance was somewhat eerie or unnatural.	(Song and Shin, 2024; Zhang T. et al., 2025), Expert interviews
	While watching, I felt that the visual presentation of the video was somewhat uncomfortable.	
	While watching, I experienced a sense of incongruity, as the characters looked human but not completely human.	
Perceived realism	I think the characters and their presentation in the video appear close to real life.	(Hu et al., 2024; Liu and Sun, 2025), Expert interviews
	I think the movements and overall presentation of the video appear natural and credible.	
	I think this video gives me an experience similar to footage captured in real-life filming.	
Perceived trust	I believe that the overall visuals of this AI-generated video are reliable.	(Ahmed and Aziz, 2025; Xu T. et al., 2025; Zhang T. et al., 2025)
	I believe that the visual presentation of the characters in the video is natural and trustworthy.	
	I believe that, overall, the visual effects of this video are worthy of trust.	
Usage intention	I am willing to continue watching similar AI-generated videos.	(Al-Adwan et al., 2023; García de Blanes Sebastián et al., 2022; Jiang et al., 2025; Liu and Sun, 2025)
	I am willing to use AI-generated videos as materials when needed.	
	I am willing to recommend this type of AI-generated video to others.	

The experiment was conducted via an online survey platform, with videos presented through embedded video players. Participants were required to fully watch each video before completing the corresponding questionnaire, continuing this process until all three videos had been viewed and evaluated.

### 3.3 Pilot study

To assess the quality of the questionnaire measures and the effectiveness of the video stimulus manipulation, a small-scale pilot study was conducted prior to the formal experiment. A total of 88 valid questionnaires were collected. Each participant viewed three videos (low, medium, and high hallucination levels), yielding 264 valid observations. The results indicated good scale reliability across all dimensions, with Cronbach's α ranging from 0.888 to 0.944. The Kaiser–Meyer–Olkin (KMO) value was 0.923, and Bartlett's test of sphericity was significant (*p* < 0.001). All factor loadings exceeded 0.70, composite reliability (CR) ranged from 0.933 to 0.965, and average variance extracted (AVE) ranged from 0.823 to 0.901. These results demonstrate satisfactory convergent and discriminant validity of the measurement scales (Cheung et al., 2024; Eisinga et al., 2013; Izah et al., 2023; Moussaïd et al., 2013; Sobti, 2019; Tian and Hong, 2012).

To verify the effectiveness of AI hallucination manipulation, differences in perceived AI hallucinations (AIH) across the three video stimuli were examined. The results revealed significant differences among hallucination levels (*F* = 116.66, *p* < 0.001). A linear trend test was also significant (*F* = 205.33, *p* < 0.001), indicating a stable increasing pattern in participants' perceived hallucination intensity from low to medium to high conditions. Further pairwise comparisons showed that all differences between hallucination levels were statistically significant (all *p* < 0.05). These findings confirm that the manipulation of AI hallucination intensity was successful at the perceptual level and that the stimuli exhibited clear discriminability.

Participants in the pilot study generally reported smooth video playback, clear differences in hallucination levels, and well-defined questionnaire items. Based on the pilot results, minor adjustments were made to the stimulus presentation order and questionnaire wording, after which the study proceeded to the formal experimental phase.

### 3.4 Formal experiment

The formal experiment was conducted between November and December 2025. The questionnaire was distributed via the Tencent Questionnaire platform, with IP addresses and device information used to screen for duplicate responses. A total of 408 valid samples were obtained (see Table 2). Among the participants, 72.5% were female, 89.2% were aged 18–24, and 93.6% had professional backgrounds related to design, visual media, or film and television.

**Table 2:** Basic demographic information.

**Characteristics**	**Categories**	***N***	**%**
Gender	Male	112	27.5
	Female	296	72.5
Age group	≤ 18 years	26	6.4
	18–24 years	364	89.2
	25–30 years	8	2.0
	31–40 years	10	2.5
	≥40	0	0
Professional background	Design, visual media, or film-related fields	382	93.6
	Non-related fields	26	6.4
Total		408	100.0

Each participant viewed three videos representing different levels of AI hallucination (low, medium, and high) and provided ratings across five dimensions. In total, 1,224 rating records were collected, corresponding to one evaluation per hallucination level for each participant. Given the within-subject three-condition design, the analyses were divided into two components: mechanism testing and condition difference testing. Mechanism testing aimed to examine how individuals' subjective perceptions of hallucination intensity (AIH) influence perceived trust (PT) through uncanny valley eeriness (UVE) and perceived realism (PR), and how these effects further translate into behavioral intention (UI). Accordingly, in the PLS-SEM analysis, participants served as the unit of analysis. Construct scores across the three video conditions were aggregated within individuals (i.e., averaged across conditions), resulting in an individual-level dataset (*N* = 408) for estimating the measurement and structural models. This approach provided more stable overall evaluations and mitigated potential biases arising from dependency among repeated observations.

Condition difference testing, by contrast, retained the repeated-measures structure and employed one-way repeated-measures analysis of variance to examine mean differences across low, medium, and high hallucination conditions. Together, these two analytical approaches addressed whether the proposed psychological mechanisms were supported and whether the experimental manipulation produced significant condition-based differences, with results from both analyses mutually reinforcing the study's conclusions.

## 4 Data analysis and results

To systematically test the proposed trust formation model and its underlying mechanisms, this chapter applies a multi-stage analytical procedure. First, the reliability and validity of the measurement model are evaluated to ensure that the scales meet the requirements for structural model testing. Next, grounded in the Stimulus–Organism–Response (S–O–R) framework, a partial least squares structural equation model (PLS-SEM) is constructed to examine the effects of the external stimulus (AI hallucinations) on emotional, cognitive, and psychological responses and, ultimately, on behavioral outcomes. Mediation analyses are further conducted to verify the transmission role of trust between psychological processing and behavioral intention. In addition, analysis of variance is used to compare mean differences across hallucination-level conditions to assess the effectiveness of the experimental manipulation and to complement the interpretation of AI hallucination effects from a between-condition perspective. The results are reported in sequence below.

### 4.1 Reliability and validity assessment

To examine the reliability and validity of the measurement model, this study adopts standard measurement-model evaluation procedures within the SEM framework. First, for indicator reliability, standardized factor loadings are used to assess the extent to which each observed variable explains its corresponding latent construct. As shown in Table 3, all item loadings are well above the recommended threshold of 0.70 (Moussaïd et al., 2013; Sobti, 2019). Specifically, loadings range from 0.911 to 0.937 for AIH, 0.758 to 0.945 for UVE, 0.884 to 0.937 for PR, 0.958 to 0.967 for PT, and 0.878 to 0.933 for UI, indicating that the indicators adequately represent their intended constructs.

**Table 3:** Reliability and convergent validity of the measurement model.

**Construct**	**Items**	**Factor loadings**	**Cronbach's alpha**	**CR**	**AVE**
AIH	AIH1	0.937	0.922	0.950	0.865
	AIH2	0.942			
	AIH3	0.911			
UVE	UVE1	0.934	0.860	0.913	0.780
	UVE2	0.945			
	UVE3	0.758			
PR	PR1	0.884	0.908	0.942	0.845
	PR2	0.936			
	PR3	0.937			
PT	PT1	0.958	0.959	0.974	0.925
	PT2	0.967			
	PT3	0.960			
UI	UI1	0.921	0.898	0.936	0.830
	UI2	0.878			
	UI3	0.933			

Regarding internal consistency, Cronbach's α values for the latent variables range from 0.860 to 0.959 (Izah et al., 2023), and composite reliability (CR) values range from 0.913 to 0.974, exceeding the recommended criterion of 0.70 (Cheung et al., 2024). These results suggest strong and stable internal consistency across constructs.

For convergent validity, average variance extracted (AVE) is computed. The AVE values for AIH, UVE, PR, PT, and UI are 0.865, 0.780, 0.845, 0.925, and 0.830, respectively, all exceeding the 0.50 benchmark (Cheung et al., 2024), thereby supporting adequate convergent validity.

It is important to emphasize that, prior to constructing the PLS-SEM structural model, this study performed within-individual aggregation of each participant's ratings across the low-, medium-, and high-hallucination conditions. The exceptionally strong indicators of internal consistency and convergent validity reported in Table 3 provide compelling post hoc statistical justification for the appropriateness of this aggregation procedure. These results demonstrate that the within-individual data aggregation did not introduce systematic random noise or distort the underlying construct structure. Rather, it effectively extracted a stable and consistent trait-level psychological baseline across varying levels of visual distortion intensity.

Furthermore, this aggregation strategy enabled the SEM analysis to focus on the overall S–O–R psychological transmission mechanism operating across different hallucination intensities. In contrast, the differentiated effects associated with specific levels of hallucination intensity were examined separately through subsequent analyses of variance (ANOVA). Together, these two analytical approaches form a rigorous and complementary explanatory framework for understanding the proposed mechanisms.

Discriminant validity is further confirmed using cross-loadings and the Fornell–Larcker criterion. As shown in Table 4, each item loads higher on its assigned construct than on other constructs. Table 5 additionally indicates that the square roots of AVE for AIH, UVE, PR, PT, and UI (0.930, 0.883, 0.919, 0.962, and 0.911, respectively) are greater than the corresponding inter-construct correlations. Overall, the measurement model demonstrates satisfactory reliability, convergent validity, and discriminant validity, supporting subsequent structural model analysis.

**Table 4:** Cross-loadings of measurement items.

**Items**	**AIH**	**UVE**	**PR**	**PT**	**UI**
AIH1	**0.937**	0.516	−0.344	−0.365	−0.312
AIH2	**0.942**	0.544	−0.347	−0.368	−0.341
AIH3	**0.911**	0.491	−0.301	−0.332	−0.273
UVE1	0.515	**0.934**	−0.181	−0.227	−0.161
UVE2	0.581	**0.945**	−0.234	−0.279	−0.221
UVE3	0.330	**0.758**	−0.051	−0.118	−0.068
PR1	−0.301	−0.130	**0.884**	0.715	0.593
PR2	−0.321	−0.192	**0.936**	0.824	0.656
PR3	−0.358	−0.203	**0.937**	0.856	0.700
PT1	−0.355	−0.227	0.829	**0.958**	0.753
PT2	−0.363	−0.247	0.853	**0.967**	0.759
PT3	−0.385	−0.244	0.833	**0.960**	0.780
UI1	−0.316	−0.224	0.727	0.808	**0.921**
UI2	−0.309	−0.111	0.564	0.639	**0.878**
UI3	−0.285	−0.154	0.630	0.707	**0.933**

Bold values indicate the highest loading of each measurement item on its corresponding construct.

**Table 5:** Discriminant validity of the measurement model (Fornell-Larcker criterion).

	**AIH**	**UVE**	**PR**	**PT**	**UI**
AIH	**0.930**				
UVE	0.557	**0.883**			
PR	−0.357	−0.193	**0.919**		
PT	−0.382	−0.249	0.872	**0.962**	
UI	−0.333	−0.183	0.709	0.794	**0.911**

Bold values represent the square root of the average variance extracted (AVE).

Finally, to rule out the potential influence of common method bias (CMB) arising from the data collection process, this study conducted Harman's single-factor test as a diagnostic procedure. All measurement items corresponding to the core latent constructs were entered into an unrotated principal component analysis (PCA). The results indicated that the largest single factor extracted accounted for only 47.32% of the total variance, which is strictly below the critical threshold of 50%. Therefore, the findings suggest that serious common method bias is not present in the data.

### 4.2 Structural equation modeling and path analysis

Based on the S–O–R framework, PLS-SEM is employed to test the relationships among the external stimulus (S), organismic responses (O), and behavioral outcomes (R). As reported in Table 6, all hypothesized paths are statistically significant (*T* > 5, *p* < 0.001), indicating strong statistical robustness of the model (Wang et al., 2023).

**Table 6:** Hypothesis testing results of the structural model.

**Hypothesis**	**Path**	**β**	**SE**	***T* statistics**	***p-values***	**Supported**
H1	AIH → UVE	0.557	0.030	18.726	< 0.001^*^	Yes
H2	AIH → PR	−0.357	0.029	12.123	< 0.001^*^	Yes
H3	AIH → PR → PT	−0.305	0.025	12.37	< 0.001^*^	Yes
H4	AIH → UVE → PT	−0.047	0.008	5.928	< 0.001^*^	Yes
H5	UVE → PT	−0.084	0.013	6.406	< 0.001^*^	Yes
H6	UVE → PT → UI	−0.067	0.010	6.452	< 0.001^*^	Yes
H7	PR → PT	0.856	0.014	62.446	< 0.001^*^	Yes
H8	PR → PT → UI	0.68	0.019	35.299	< 0.001^*^	Yes
H9	PT → UI	0.794	0.017	47.722	< 0.001^*^	Yes
H10	AIH → UVE → PT → UI	−0.037	0.006	5.954	< 0.001^*^	Yes
H11	AIH → PR → PT → UI	−0.242	0.020	12.157	< 0.001^*^	Yes

^*^p < 0.001.

At the stimulus stage (S), AI hallucination intensity exerts significant effects on emotional and cognitive processing. Specifically, AIH has a significant positive effect on uncanny valley eeriness (AIH → UVE: β = 0.557, *T* = 18.726) and a significant negative effect on perceived realism (AIH → PR: β = −0.357, *T* = 12.123). These results indicate that higher hallucination intensity is associated with stronger discomfort and weaker judgments of realism.

At the organism stage (O), the emotional and cognitive variables form a clear psychological transmission structure. Uncanny valley eeriness negatively affects perceived trust (UVE → PT: β = −0.084, *T* = 6.406), whereas perceived realism has a strong and largest positive effect on perceived trust (PR → PT: β = 0.856, *T* = 62.446). In addition, two bootstrapped indirect effects are significant: AIH affects perceived trust indirectly via uncanny valley eeriness (AIH → UVE → PT: β = −0.047, *T* = 5.928) and via perceived realism (AIH → PR → PT: β = −0.305, *T* = 12.370).

At the response stage (R), perceived trust significantly and positively predicts behavioral intention (PT → UI: β = 0.794, *T* = 47.722), and two complete mediation pathways are supported: PR → PT → UI (β = 0.680, *T* = 35.299) and UVE → PT → UI (β = −0.067, *T* = 6.452). Ultimately, the two full S–O–R chains are validated—AIH → UVE → PT → UI (β = −0.037, *T* = 5.954) and AIH → PR → PT → UI (β = −0.242, *T* = 12.157)—demonstrating that AI hallucinations influence behavioral intention through increased emotional discomfort and reduced perceived realism, which shape trust judgments in a typical S–O–R psychological processing pattern.

To assess the model's explanatory and predictive performance, this study further examines *R*^2^, adjusted *R*^2^ (Adj. *R*^2^), and *Q*^2^ for endogenous constructs (Table 7). Following the criteria proposed by (Hair et al. 2011), UVE shows an *R*^2^ of 0.310, indicating an acceptable level of explained variance. PR has a lower *R*^2^ of 0.127, suggesting that perceived realism may be influenced by additional external factors not captured in the model. In contrast, perceived trust (PT; *R*^2^ = 0.767) demonstrates high explanatory power, and behavioral intention (UI; *R*^2^ = 0.631) reaches a moderate-to-high level, highlighting the central role of trust in the behavioral formation mechanism. The close alignment between Adj. R^2^ and R^2^ values suggests stable model estimation without signs of abnormal fit.

**Table 7:** R^2^ and Q^2^ values of endogenous constructs.

**Construct**	***R^2^***	**Adj. *R^2^***	***Q^2^***
UVE	0.310	0.309	0.307
PR	0.127	0.126	0.125
PT	0.767	0.767	0.143
UI	0.631	0.631	0.107

For predictive relevance, *Q*^2^ values are calculated using the blindfolding procedure. All *Q*^2^ values are greater than zero (UVE = 0.307, PR = 0.125, PT = 0.143, UI = 0.107), indicating satisfactory out-of-sample predictive relevance of the model (Ibrahim and Mas'ud, 2016). Overall, the proposed model shows strong explanatory power for emotional and behavioral outcome variables, demonstrates stable psychological processing paths, and provides a reliable basis for subsequent path interpretation.

### 4.3 Comparative analysis across AI hallucination levels

To examine the effects of different levels of AI hallucinations on users' perceptual and behavioral responses, hallucination intensity was treated as the independent variable, and analyses of variance were conducted on perceived AI hallucinations (AIH), uncanny valley eeriness (UVE), perceived realism (PR), perceived trust (PT), and behavioral intention (UI). As shown in Table 8, all five core variables exhibited significant differences across the three hallucination conditions (*F* = 21.353–288.154, *p* < 0.001), indicating that hallucination level has a significant impact on users' psychological processing and behavioral responses.

**Table 8:** Comparison across AI hallucination levels (repeated–measures ANOVA).

**Variable**	**Hallucination Level (M** ± **SD, within–subject**, ***N*** = **408)**			***F***	***p***
	**Low hallucination**	**Medium hallucination**	**High hallucination**		
AIH	4.45 ± 1.58	6.32 ± 1.41	6.59 ± 1.12	288.154	< 0.001^*^
UVE	5.42 ± 1.45	5.93 ± 1.73	6.11 ± 1.50	21.353	< 0.001^*^
PR	3.64 ± 1.49	2.23 ± 1.73	2.11 ± 1.70	109.159	< 0.001^*^
PT	3.33 ± 1.50	2.01 ± 1.70	1.92 ± 1.64	97.734	< 0.001^*^
UI	3.62 ± 1.56	2.25 ± 1.68	2.26 ± 1.68	94.419	< 0.001^*^

^*^p < 0.001.

Results are based on one–way repeated–measures ANOVA (within–subject design, N = 408).

Specifically, AIH scores increased significantly with hallucination intensity (M_low = 4.45 ± 1.58; M_mid = 6.32 ± 1.41; M_high = 6.59 ± 1.12), suggesting that higher levels of generative distortion make visual anomalies more salient and easier for users to detect. In contrast, perceived realism and perceived trust declined significantly as hallucination intensity increased. PR decreased from 3.64 to 2.11, and PT decreased from 3.33 to 1.92 (both *F* > 90, *p* < 0.001), indicating that higher hallucination intensity consistently weakens users' judgments of authenticity and credibility in AI-generated videos.

In addition, uncanny valley eeriness and behavioral intention showed parallel patterns. Under high-hallucination conditions, users' discomfort increased significantly, while their willingness to use and adopt the videos declined markedly (both *p* < 0.001). When video content deviates from real-world structural rules and exhibits noticeable distortions, users are more likely to experience emotional distancing and to reduce their subsequent usage intentions.

Notably, the *F-values* for AIH (*F* = 288.154) and PR (*F* = 109.159) were relatively large, reflecting more pronounced mean differences among the three hallucination conditions and further supporting the perceptual discriminability of the hallucination intensity manipulation. These results are primarily attributable to substantial between-group differences combined with relatively small within-group variability, leading to significantly greater between-group variance. This pattern indicates that user perceptual responses elicited by different hallucination intensities are characterized by clear and stable distributions, further validating the effectiveness of the experimental manipulation and highlighting the critical role of visual distortion in credibility judgments of AI-generated videos.

## 5 Discussion

### 5.1 Validating the S–O–R mechanism: psychological transmission pathways through which AI hallucinations influence behavioral intention via emotion, cognition, and trust

The results demonstrate that AI hallucinations, as an external stimulus (S), significantly activate multiple organism-level psychological responses, including emotional reactions (uncanny valley eeriness), cognitive evaluations (perceived realism), and trust judgments (perceived trust). Through these interconnected psychological processes at the organism level, AI hallucinations further shape users' subsequent behavioral responses. At the empirical level, the overall path structure exhibits a clear “stimulus–organism–response” transmission pattern. The structural equation modeling results show that all key paths reach a high level of statistical significance (*p* < 0.001), thereby confirming the applicability and explanatory power of the S–O–R model in the context of AI-generated videos. These findings indicate that users' behavioral responses in AI video contexts are not driven directly by the technical attributes of generative systems themselves, but rather are formed gradually through emotional experiences, cognitive evaluations, and trust judgments of visual cues.

From the perspective of specific pathways, higher levels of AI hallucinations significantly intensify users' feelings of eeriness and discomfort, thereby triggering typical uncanny valley responses. At the same time, increased hallucination intensity markedly weakens users' cognitive evaluations of the authenticity and consistency of AI-generated video content. These two types of psychological responses correspond to the emotional and cognitive processing components of the organism stage and jointly influence users' trust judgments. These findings not only support prior research on the uncanny valley effect, which suggests that visual stimuli that are “highly realistic yet not fully natural” are more likely to evoke negative emotional responses (Duong and Vo, 2025; Weisman and Peña, 2021), but also align with studies arguing that visual distortions disrupt cognitive consistency in human–computer interaction, thereby undermining trust formation (Carrasco-Farré, 2022; Sun et al., 2024).

Further path analysis reveals that trust plays a critical mediating and integrative role in this psychological transmission process. Specifically, when AI hallucinations induce emotional discomfort or reduce perceived realism, users' trust judgments decline accordingly; in turn, lower trust levels significantly suppress users' intentions to use and adopt AI-generated videos. As a result, emotional reactions and cognitive biases are ultimately translated into observable behavioral outcomes. This finding is consistent with prior research showing that when AI-generated videos are perceived as credible and sufficiently realistic in terms of visual presentation, narrative coherence, or emotional cues, perceived trust increases significantly and further promotes continued use and engagement behaviors (Aziz et al., 2025; Duong and Vo, 2025).

From an overall mechanistic perspective, this study demonstrates that AI hallucinations do not directly influence users' trust judgments or behavioral outcomes. Instead, their effects are indirectly transmitted through a psychological pathway composed of emotional responses, cognitive evaluations, and trust judgments, which together shape users' response patterns. Theoretically, these findings not only validate the core S–O–R assumption of “stimulus–psychological processing–behavioral response” in the context of generative AI, but also extend the application boundaries of uncanny valley theory in AI visual content and human–computer interaction research. In doing so, the study deepens understanding of the mechanisms linking visual distortion, psychological transmission, and user responses.

### 5.2 Comparing organism-stage psychological variables: the behavioral predictive roles of emotion, cognition, and trust

After confirming the overall S–O–R mechanism, this study further focuses on the differential predictive roles of key psychological variables within the organism stage in shaping behavioral intention. Specifically, it systematically compares the functional contributions and relative importance of emotional responses (uncanny valley eeriness), cognitive evaluations (perceived realism), and psychological judgments (perceived trust) in the formation of users' behavioral intentions.

The structural model results reveal significant differences in both the pathways and effect strengths of these psychological variables in predicting behavior. First, perceived realism, as a core cognitive evaluation, exerts a strong and the most substantial positive effect on perceived trust (β = 0.856, *p* < 0.001), indicating that users' judgments regarding the authenticity and consistency of AI-generated videos constitute a critical foundation for trust formation. By contrast, uncanny valley eeriness influences trust through a negative pathway (β = −0.084, *p* < 0.001), suggesting that discomfort triggered by visual incongruity undermines trust tendencies, although its effect magnitude is relatively modest and functions more as a negative interfering factor.

Further analysis indicates that perceived trust occupies a central position in behavioral prediction. Perceived trust has a significant and strong direct effect on behavioral intention (β = 0.794, *p* < 0.001), with an effect size clearly exceeding the direct or indirect influences of emotional and cognitive variables. This result suggests that both emotional reactions and cognitive evaluations must be mediated through trust in order to be translated into users' intentions to use, adopt, or share AI-generated videos.

From an overall behavioral prediction structure, the findings reveal a clear functional hierarchy within the organism stage. Emotional responses primarily serve a negative interference role, cognitive evaluations provide essential input for trust judgments, and trust acts as the core hub linking internal psychological processing with external behavioral responses. These results not only deepen understanding of user decision-making mechanisms in AI-generated video contexts, but also empirically underscore the pivotal role of trust in the acceptance of generative AI content, offering important implications for trust-oriented design and system optimization.

### 5.3 Differential effects of AI hallucination intensity on emotion, cognition, and trust

Following the confirmation of the overall mechanism through which AI hallucinations influence user behavior via emotion, cognition, and trust, this study further examines whether different levels of AI hallucination intensity produce systematic variations in key psychological variables. The analysis of variance results indicate significant differences across hallucination levels in uncanny valley eeriness, perceived realism, and perceived trust (all *p* < 0.001), demonstrating that hallucination intensity, as a critical visual stimulus condition, significantly shapes users' emotional experiences, cognitive judgments, and trust evaluations.

At the emotional level, uncanny valley eeriness increases significantly with rising AI hallucination intensity (*F* = 21.353, *p* < 0.001). Specifically, users report relatively mild discomfort under low-hallucination conditions, whereas eeriness and psychological incongruity become more pronounced under medium and high hallucination conditions (M_low = 5.42; M_mid = 5.93; M_high = 6.11). This finding suggests that users are highly emotionally sensitive to visual anomaly cues, with higher hallucination intensity more likely to trigger negative emotional responses. The result is consistent with prior studies indicating that AI hallucinations distort information and evoke uncanny valley reactions (Sun et al., 2024; Zhou and Lu, 2025).

At the cognitive level, perceived realism declines significantly as hallucination intensity increases (*F* = 109.159, *p* < 0.001). Videos with low hallucination levels receive substantially higher realism evaluations than those with medium or high hallucination levels (M_low = 3.64; M_mid = 2.23; M_high = 2.11). This pattern indicates that when AI-generated videos exhibit structural distortions, discontinuous movements, or semantic inconsistencies, users' cognitive judgments of authenticity and consistency are markedly compromised. Compared with emotional responses, the decline in perceived realism is more pronounced, which further explains why perceived realism shows stronger predictive power in trust formation in the structural model analysis. This finding aligns with prior research demonstrating that AI hallucinations undermine realism perception (Carrasco-Farré, 2022; Sun et al., 2024).

At the trust evaluation level, perceived trust likewise decreases steadily with increasing AI hallucination intensity (*F* = 97.734, *p* < 0.001). Videos under low-hallucination conditions are more likely to be perceived as stable and reliable, with trust levels significantly higher than those under medium and high hallucination conditions (M_low = 3.33; M_mid = 2.01; M_high = 1.92). These results indicate that as visual distortions and logical anomalies accumulate, users' overall credibility judgments of AI-generated videos are substantially weakened, thereby undermining the psychological basis for subsequent adoption and use. This finding is consistent with prior studies suggesting that AI hallucinations indirectly erode user trust through information distortion (Aziz et al., 2025; Zhang et al., 2025b).

Overall, different levels of AI hallucinations produce consistent and significant effects across emotional, cognitive, and trust-related dimensions. By examining hallucination intensity as a stimulus condition, these results complement the preceding S–O–R path analyses and behavioral prediction models, further demonstrating that AI hallucinations not only determine whether psychological responses occur, but also regulate their magnitude and cumulative impact. Together, these findings provide more comprehensive empirical support for understanding the mechanisms underlying credibility formation in AI-generated videos.

## 6 Conclusion

### 6.1 Summary of core findings

Grounded in the Stimulus–Organism–Response (S–O–R) framework, this study systematically examined the psychological transmission mechanisms through which AI hallucinations in AI-generated videos trigger a user trust crisis. The findings demonstrate that AI hallucinations are not merely technical flaws but powerful visual stimuli that evoke deep psychological rejection. Within the organismic stage, perceived realism—linked to fundamental logical coherence—exerted a dominant influence on trust formation, surpassing the transient emotional discomfort associated with uncanny valley responses. Furthermore, the destructive effects of different hallucination intensities were not linearly progressive but characterized by tolerance thresholds and cliff-like collapse effects. Perceived trust functioned as the central psychological hub ultimately determining users' adoption intentions toward AI-generated video.

### 6.2 Theoretical implications

This study contributes three major theoretical advances to interdisciplinary research in generative AI and human–computer interaction:

First, it extends the application boundaries of uncanny valley theory and the S–O–R model. By shifting the uncanny valley effect from static robotic entities to dynamic generative AI video contexts, the study constructs a comprehensive theoretical chain of visual distortion–psychological processing–behavioral response, offering a structured framework for understanding visual trust formation in AIGC.

Second, it clarifies the asymmetric roles of cognition and emotion in trust formation. Whereas, prior research often assumes equal contributions, this study empirically demonstrates a cognition-dominant pattern in AI video contexts. Perceived realism (cognitive foundation) plays a decisively stronger role than uncanny valley emotion (affective disturbance), thereby refining dual-process accounts of AI trust.

Third, it introduces a nonlinear disruption perspective on visual stimuli. The findings reveal the moderating role of hallucination intensity and uncover a dynamic pattern of psychological resource accumulation and threshold collapse, deepening theoretical understanding of how visual distortion systematically dismantles trust.

### 6.3 Practical implications

The findings provide clear guidance for the development, optimization, and governance of AI video generation systems:

First, they establish a trust-oriented priority for system optimization. Because moderate- and high-intensity hallucinations trigger cliff-like breakdowns in cognition and trust, developers should prioritize detection and interception mechanisms targeting structural distortions and semantic inconsistencies. Ensuring coherence in underlying physical and logical rules is more critical than merely enhancing the visual quality of minor details.

Second, the study offers a psychologically informed framework for evaluating AIGC content quality. Visual realism does not necessarily equate to psychological credibility. Industry standards for AI-generated video assessment should incorporate user-cantered psychological thresholds—such as uncanny valley sensitivity and realism tolerance—to guide the production of visually credible content aligned with human cognitive expectations.

### 6.4 Limitations and future research

Despite its systematic contributions, this study has several limitations that suggest directions for future research:

First, the demographic concentration of the sample may limit generalizability. Participants were primarily young individuals with design or visual media backgrounds (89.2% aged 18–24; 72.5% female). As early adopters and high-frequency users of AIGC tools with strong visual literacy, they are particularly sensitive to subtle structural distortions and semantic anomalies. This provided a highly sensitive context for testing the proposed mechanism and ensured strong internal validity. Moreover, the within-subject design effectively controlled for between-subject variance (e.g., gender and age), enhancing robustness. However, this heightened visual sensitivity may constrain generalizability. Broader populations without specialized training may display greater tolerance for minor visual flaws and may not detect low-intensity hallucinations. Consequently, the strong emotional and trust breakdown effects observed here may be attenuated in general populations. Future research should incorporate more diverse age groups and cross-cultural samples to further examine external validity.

Second, limitations in stimulus materials remain. The stimuli were derived from selected mainstream AI video generation models, and the within-subject design may introduce intra-individual correlations. Future studies could incorporate more diverse generative models—such as comparisons between physics-based simulation engines and diffusion models—and apply hierarchical linear modeling for refined statistical control.

Third, the range of psychological variables could be expanded. While this study focused on uncanny valley emotion and perceived realism, future research may integrate perceived risk, cognitive load, and AI literacy to construct a more comprehensive decision-processing network in AIGC contexts.

Fourth, limitations exist in the data analysis strategy. In PLS-SEM testing, repeated measures were aggregated to extract stable psychological baselines. Although effective, this approach may obscure within-subject variance across hallucination conditions. Future studies may adopt multilevel SEM to preserve stimulus-level variation and more precisely model condition-specific psychological mechanisms.

### 6.5 Concluding remarks

As generative AI continues to reshape the visual media landscape, the transition of AI video from “technically functional” to “socially trustworthy” has become a central bottleneck for large-scale adoption. Moving beyond purely technical evaluation, this study provides in-depth psychological evidence explaining how AI hallucinations erode trust by undermining perceived realism and activating uncanny valley responses. By illuminating this “trust black box” in AI-generated content, the findings offer both theoretical clarity and practical direction for designing future human–AI interaction systems grounded in logical coherence and visual credibility.

## Data Availability

The raw data supporting the conclusions of this article will be made available by the authors, without undue reservation.
